# Supplementary material for: Mapping the evolution of fertility support policies in China: A content and instrumental analysis
Source: PLoS One. 2025 Oct 9;20(10):e0332137. doi: 10.1371/journal.pone.0332137 (PMC12510515; doi:10.1371/journal.pone.0332137)
Supplement: S1 Appendix — (ZIP) [file pone.0332137.s001.zip › S1 Appendix. 226 original policy documents/69-国务院关于今年以来国民经济和社会发展计划执行情况的报告(2019)(FBM-CLI-2-335248).doc]

国务院关于今年以来国民经济和社会发展计划执行情况的报告(2019)

**发布部门：** [国务院](https://www.pkulaw.com/law/chl?Aggs.IssueDepartment=5)

**发布日期：**2019.08.23

**实施日期：**2019.08.23

**时效性：** [现行有效](https://www.pkulaw.com/law/chl?Aggs.TimelinessDic=01)

**效力级别：** [国务院规范性文件](https://www.pkulaw.com/law/chl?Aggs.EffectivenessDic=XC0203)

**法规类别：** [国家计划](https://www.pkulaw.com/law/chl?Aggs.Category=02404) [营商环境优化](https://dobusiness.pkulaw.com/)

国务院关于今年以来国民经济和社会发展计划执行情况的报告
--2019年8月23日在第十三届全国人民代表大会常务委员会第十二次会议上
国家发展和改革委员会主任　何立峰

全国人民代表大会常务委员会：
　　受国务院委托，我向全国人大常委会报告今年以来国民经济和社会发展计划执行情况，请审议。
　　今年上半年，面对国内外风险挑战明显增多的复杂局面，在以习近平同志为核心的党中央坚强领导下，各地区各部门认真贯彻落实党中央、国务院各项决策部署，保持战略定力，坚持稳中求进工作总基调，坚持新发展理念，坚持推动高质量发展，坚持以供给侧结构性改革为主线，坚持深化市场化改革、扩大高水平开放，认真执行十三届全国人大二次会议审议批准的《政府工作报告》和2019年国民经济和社会发展计划，落实全国人大财政经济委员会的审查意见，贯彻落实“巩固、增强、提升、畅通”八字方针，统筹推进稳增长、促改革、调结构、惠民生、防风险、保稳定工作，有力有效应对经贸摩擦，扎实办好自己的事，加大“六稳”工作力度，各项工作取得积极成效。上半年国内生产总值增长6.3%，城镇新增就业737万人，全国城镇调查失业率保持在5%左右，居民消费价格上涨2.2%，外汇储备保持在3万亿美元以上，实物量指标与经济增长基本匹配。总的来看，我国经济延续总体平稳、稳中有进的运行态势，主要宏观经济指标处在合理区间，供给侧结构性改革持续推进，改革开放继续深化，就业比较充分，精准脱贫有序推进，生态环境持续改善，创新动能不断集聚，市场主体活力不断增强，人民生活水平和质量继续提高，推动高质量发展的积极因素增多，保持了经济社会持续健康发展的大局。
　　（一）狠抓宏观政策落地见效，经济运行保持稳定。落实《政府工作报告》和计划报告总体要求和宏观经济政策取向，坚持不搞“大水漫灌”，注重打好宏观调控“组合拳”，着力提升政策的前瞻性、精准性和实效性，超前谋划出台一批政策，提前部署一批建设项目和任务，提早下达部分财政转移支付和地方政府新增债务限额，对稳定经济运行和提振市场信心发挥了重要作用。积极的财政政策加力提效，深化增值税改革、小微企业普惠性减税、降低社保费率、设立个人所得税专项附加扣除、清理规范政府性基金等更大规模减税降费措施全面实施，上半年累计新增减税降费11709亿元，财政预算下达和地方政府债券发行进度加快，全国一般公共预算收入和支出分别增长3.4%、10.7%，新增地方政府债券发行21765亿元。稳健的货币政策松紧适度，综合实施下调存款准备金率、定向降准和中期借贷便利等措施，保持市场流动性合理充裕，6月末广义货币M2余额同比增长8.5%，社会融资规模存量增长10.9%，与国内生产总值名义增速基本匹配。以永续债为突破口，推动银行多渠道补充资本。完善金融服务民营企业、小微企业支持政策体系，深化民营和小微企业金融服务综合改革试点，普惠型小微企业贷款余额较年初较快增长、利率水平持续下降。就业优先政策全面发力，中央财政安排就业补助资金支持落实各项就业创业补贴政策，加大失业保险稳岗返还力度，出台职业技能提升行动方案，全面启动运用1000亿元失业保险基金开展大规模职业技能培训工作，积极发挥“双创”对就业的带动支撑作用，着力做好重点群体就业创业工作，就业形势保持总体稳定。
　　（二）着力推进“放管服”改革，营商环境持续优化。按照《政府工作报告》和计划报告关于激发市场主体活力、着力优化营商环境等要求，各部门以打造市场化、法治化、国际化营商环境为着力点，多措并举推动解决影响企业创新发展的痛点难点堵点问题，缓解实体企业困难，激发微观主体活力。继续加大“放”的力度，开展[市场准入负面清单](https://www.pkulaw.com/chl/d8135b05437494cebdfb.html)（2019年版）修订工作，推动“非禁即入”普遍落实，工程建设项目审批制度改革在全国推开，企业开办时间压缩至8.5个工作日以内，注销流程和手续进一步压减。行业协会商会与行政机关脱钩改革全面推开。注重优化“管”的方式，改进和加强竞争执法，社会信用体系建设深入开展，“双随机、一公开”跨部门联合监管持续推进，信用监管和“互联网＋监管”改革加快推行，政府机构失信问题专项治理有序开展。努力提升“服”的实效，建立健全企业家参与涉企政策制定机制，国家政务服务平台上线试运行，在水、气、暖等公用事业领域推行APP（应用程序）办事、移动支付，压减办理用电业务时间，发展“互联网＋教育”、“互联网＋医疗”等，优化口岸营商环境。以改革推动降低涉企收费，全面落实一般工商业电价平均再降10%的目标任务，进一步清理规范行政事业性收费和政府定价经营服务性收费，推动降低中小企业宽带资费、移动网络流量平均资费，取消或降低一批铁路、公路、港口、民航收费，加快取消全国高速公路省界收费站，清理拖欠民营企业和中小企业账款取得积极进展。上半年，日均新登记企业1.94万户、同比增长7.1%。
　　（三）加快结构调整升级步伐，创新创业创造向纵深拓展。按照《政府工作报告》和计划报告关于坚持创新引领、培育壮大新动能等要求，各部门聚焦振兴实体经济，坚持创新引领发展，努力促进新旧动能接续转换。农业生产保持稳定，一二三产业融合发展势头良好，高标准农田建设持续推进，种养结构进一步调整优化，夏收粮油再获丰收，夏粮总产量14174万吨、增产293万吨，优质产品占比提高，秋粮苗情与常年大体相当，采取有效措施防控非洲猪瘟疫情、稳定生猪市场供应。产业转型升级持续推进，出台实施促进中小企业健康发展的指导意见，积极巩固钢铁、煤炭等重点行业去产能成效，加快推进“僵尸企业”处置工作，进一步完善降成本、债转股、市场主体退出等政策，固定资产加速折旧优惠政策适用范围扩大至全部制造业。上半年，规模以上工业增加值增长6%，其中私营工业企业增加值增长8.7%、同比有所加快，制造业技术改造投资增长13.1%。积极推动服务业高质量发展，促进先进制造业和现代服务业深度融合，上半年服务业增加值增长7%。科技支撑和新兴产业引领作用不断增强，出台加强基础研究、应用基础研究和技术创新工作的指导意见，加大首台套、首批次政策实施力度。北京、上海、粤港澳大湾区科技创新中心建设加快。大力推进人工智能、大数据等领域重大工程建设，向4家企业颁发5G牌照，继续实施集成电路设计和软件企业所得税“两免三减半”等优惠政策。上半年高技术制造业增加值增长9%、投资增长10.4%，国内发明专利授权量增长12.6%。大众创业万众创新进一步深化，双创示范基地带动作用明显增强，财税、金融、人才等扶持政策不断完善，科创板正式开板，“双创”对扩大就业、激发创新创造活力、壮大新动能、增强竞争力的积极效应加快显现。
　　（四）促进形成强大国内市场，需求结构逐步改善。按照《政府工作报告》和计划报告关于促进形成强大国内市场、持续释放内需潜力等要求，各部门紧扣更好促进供需良性循环、培育形成强大国内市场，制定出台系列政策文件并狠抓落实。优化供给推动消费平稳增长，制定实施促进重点消费品更新消费的实施方案，出台推进养老、家政服务业、婴幼儿照护服务发展的政策措施，优化“五一”劳动节休假安排。上半年，社会消费品零售总额增长8.4%，其中实物商品网上零售额增长21.6%，消费拉动经济增长的“主引擎”作用更加凸显。加大补短板有效投资力度，出台实施[政府投资条例](https://www.pkulaw.com/chl/8d88a1cd032de8b9bdfb.html)，优化政府投资结构，大力推进基础设施领域补短板，上半年下达全年中央预算内投资计划的94%，积极向金融机构提供补短板重大项目清单，允许将地方政府专项债券作为符合条件的重大项目资本金，重点在建项目和补短板工程资金保障得到加强。上半年固定资产投资增长5.8%，生态环保、教育、文化等短板领域投资分别增长48%、18.9%、22.1%。内需对上半年经济增长的贡献率达到79.3%，服务消费占居民最终消费支出的49.4%、同比提高0.6个百分点，供给体系优化对需求升级的促进作用进一步显现。
　　（五）聚焦全面建成小康社会目标任务，三大攻坚战取得新进展。按照《政府工作报告》和计划报告的要求，各部门对标全面建成小康社会任务，着力加大补短板、强弱项力度，实施精准攻坚。围绕精准脱贫难点重点开展攻坚，巩固“两不愁”成效，进一步加大解决“三保障”突出问题工作力度，在“三区三州”等深度贫困地区扎实推进产业扶贫、易地扶贫搬迁等工作；严把贫困户、贫困县乡村“退出关”，建立巩固脱贫、防止返贫长效机制，又有一批贫困县实现脱贫摘帽。生态环境质量稳定向好，持续推进打好污染防治攻坚战，印发[中央生态环境保护督察工作规定](https://www.pkulaw.com/chl/a13450644cc42819bdfb.html)，发布实施[绿色产业指导目录（2019年版）](https://www.pkulaw.com/chl/97962b817cff680ebdfb.html)，上半年单位国内生产总值能耗同比下降2.7%，全国细颗粒物平均浓度下降2.4%。重点领域风险防范取得新进展，印发实施打好防范化解重大金融风险攻坚战行动方案，依法专业高效处置个别中小银行风险，稳妥化解部分中小金融机构面临的局部性流动性风险，及时有效应对国内股市、汇市等金融市场波动，妥善处置债券违约风险及部分大型企业债务风险，持续开展金融乱象整治。有效遏制地方政府隐性债务增量，分类化解存量地方政府隐性债务风险。推动完善房地产市场平稳健康发展长效机制，落实城市主体责任制，房地产市场运行总体平稳，6月份70个大中城市中一、二、三线城市房价同比涨幅均有所回落。
　　（六）推动城乡地区协调发展，引领高质量发展的动力源不断形成。按照《政府工作报告》和计划报告关于促进区域协调发展、提高新型城镇化质量等要求，各部门围绕解决发展不平衡不充分问题，持续推进完善城乡区域相关机制和政策，发展协调性进一步提高。新型城镇化和乡村振兴体制机制更趋完善，印发[建立健全城乡融合发展体制机制和政策体系的意见](https://www.pkulaw.com/chl/d3cf3bbf007959eebdfb.html)，出台实施[培育发展现代化都市圈的指导意见](https://www.pkulaw.com/chl/d247cf138d9f2a4bbdfb.html)，全面推广第二批新型城镇化综合试点经验，城市落户限制进一步放宽。建立乡村振兴规划实施协调推进机制，省级乡村振兴战略规划全部印发实施，农村人居环境整治从典型示范转向面上推开，农村饮水安全、农网改造等重大工程项目建设进展顺利。重大区域发展战略向纵深推进，公布实施粤港澳大湾区发展规划纲要，出台长江三角洲区域一体化发展规划纲要、新时代推进西部大开发形成新格局的指导意见等重要文件，长江经济带发展、京津冀协同发展战略任务扎实推进，雄安新区由顶层设计转入实质性建设阶段，支持海南全面深化改革开放的各项政策逐步落地，有序推进东北振兴、中部崛起、东部地区率先发展工作。上半年，中部、西部地区固定资产投资分别增长9.4%和6.1%，居民人均可支配收入名义分别增长9.3%和9.1%，均快于全国平均增速。
　　（七）扎实推进改革开放，市场活力持续增强。按照《政府工作报告》和计划报告关于深化重点领域改革、推动全方位对外开放等要求，各部门聚焦关键领域和薄弱环节，不断深化改革扩大开放，完善与高质量发展相适应的体制机制。重点领域改革取得新进展，统筹推进自然资源资产产权制度改革，积极推进公共资源交易平台整合共享，健全建设用地使用权转让、出租、抵押二级市场。有序推进农村集体产权制度改革，基本完成承包地确权登记工作。确定第四批国有企业混合所有制改革试点企业，改革国有资本授权经营体制。大力推进油气管网运营机制改革。加快推动增量配电改革试点落地见效，电力现货市场建设试点全部启动。加快要素获取、准入许可、经营运行、招投标、政府采购与涉产权等政策文件和法规清理。启动涉政府产权纠纷问题专项治理行动。积极运筹全方位外交营造稳定的外部环境，习近平总书记主持第二届“一带一路”国际合作高峰论坛、亚洲文明对话大会等主场外交活动，先后出访欧亚多国并出席G20大阪峰会等重要会议，主动积极与有关国家协调立场，营造了于我有利的国际环境。李克强总理等党和国家领导同志通过出访、接待外国领导人来访、出席相关国际会议、与国际组织领导人磋商等外交活动，积极推进与相关国家的务实合作。对外经贸合作进一步拓展，推进共建“一带一路”走深走实，精准支持企业开拓多元化出口市场，加快提升通关便利化水平，抓紧推动自主降低关税工作，上半年外贸保持平稳增长，以人民币计价的进出口总值同比增长3.9%，有进出口实绩的企业数量增长5.6%；出口总值增长6.1%。出台[外商投资法](https://www.pkulaw.com/chl/6a88714068b3724dbdfb.html)和新的[鼓励外商投资产业目录](https://www.pkulaw.com/chl/2e6fab8549042788bdfb.html)、全国和自贸试验区外资准入负面清单，全面清理取消负面清单以外对外资准入的限制，有力有序推进重大外资项目落地。推动增设6个自贸试验区和上海自贸试验区新片区工作。加快推进资本市场对外开放，正式开通沪伦通。一些跨国公司加大在华投资，上半年以人民币计价实际使用外资增长7.2%，高技术行业利用外资增速高于整体水平。国际产能合作和第三方市场合作稳步推进。
　　（八）保障和改善民生工作力度加大，发展成果惠及群众。按照《政府工作报告》和计划报告关于更好保障和改善民生等要求，各部门在今年财政收支平衡压力加大的情况下，千方百计保障基本民生投入只增不减。居民收入和社会保障水平稳步提高，城乡居民增收试点工作持续推进，个税改革受益范围持续扩大，上半年全国居民人均可支配收入实际增长6.5%，略快于经济增速。城乡居民医保人均财政补助标准再提高30元，养老保险基金中央调剂比例提高至3.5%，按5%左右的比例提高退休人员基本养老金。退役军人社保接续工作有序推进。民生领域公共服务水平和质量继续提升，出台国家积极应对人口老龄化中长期规划，中国教育现代化2035及实施方案正式印发，高职院校扩招计划开始启动，扩大高职院校奖助学金覆盖面并提高补助标准，设立中等职业教育国家奖学金。健康中国行动扎实推进，重大文化设施、基层综合性文化服务中心建设有序开展。推动城镇老旧小区改造，安排中央补助资金予以支持。安全生产和食品药品安全等工作持续加强，制定出台[生产安全事故应急条例](https://www.pkulaw.com/chl/b7427510fcafaa12bdfb.html)、[深化改革加强食品安全工作的意见](https://www.pkulaw.com/chl/6402785cc5884ec3bdfb.html)、[疫苗管理法](https://www.pkulaw.com/chl/2a0405a853c122f3bdfb.html)，有序推进危化品、特种设备重大安全隐患集中排查整治等工作，及时应对处置重大生产安全事故，受地震、洪涝等自然灾害影响的群众得到妥善安置。
　　总的看，今年以来，按照党中央、国务院决策部署，我们努力应对各种困难挑战，经济运行总体平稳、结构持续优化、民生不断改善，在全球经济增速放缓的同时，我国经济仍保持稳中有进，增速在主要经济体中位居前列，展现出中国经济巨大的韧性、潜力和回旋余地。实践充分证明，党中央、国务院的决策部署是完全正确的、有力有效的。这是以习近平同志为核心的党中央坚强领导的结果，这是习近平新时代中国特色社会主义思想指导的结果，这是全党全国各族人民团结一心、艰苦奋斗的结果。
　　在经济运行总体平稳的同时，必须看到我国经济发展面临的困难挑战和不确定不稳定因素明显增多。从国际看，外部环境更趋复杂严峻，当前世界经济和贸易增速同步趋缓，近期世界银行、经合组织、世贸组织下调了今年世界经济和贸易增长预期。从国内看，经济发展面临新的风险挑战，国内经济下行压力加大，实体经济困难仍然较多，重点领域风险隐患仍存，民生领域还有不少短板。此外，一些地方生产安全事故时有发生，部分地区发生洪涝、地震等自然灾害，应急管理和防灾救灾工作任务艰巨。
　　总的来看，上半年经济延续总体平稳、稳中有进的运行态势，主要指标保持在合理区间，计划完成情况总的是好的。同时也要清醒认识到，国内一些长期存在的结构性体制性矛盾与外部冲击共同作用，经济发展面临新的风险挑战，保持经济平稳健康发展的难度有所加大。对照十三届全国人大二次会议审查批准的2019年国民经济和社会发展计划总表，当前水环境质量、棚户区改造、减贫等约束性指标进展顺利，经济增长、就业、居民收入、物价、货物进出口等主要预期目标经过努力能够完成，但完成能耗强度、空气质量等指标仍需付出更大努力。
　　做好下半年工作，意义十分重大。要更加紧密地团结在以习近平同志为核心的党中央周围，以习近平新时代中国特色社会主义思想为指导，坚持和加强党的全面领导，增强“四个意识”，坚定“四个自信”，做到“两个维护”，全面贯彻党的十九大和十九届二中、三中全会精神，按照党中央、国务院决策部署，落实中央经济工作会议精神和《政府工作报告》部署以及十三届全国人大二次会议要求，根据全国人大常委会及全国人大财经委关于经济工作的意见建议认真改进工作，坚持稳中求进工作总基调，坚持发展第一要务，着力办好自己的事，主动应对内外部环境复杂深刻变化，坚持以供给侧结构性改革为主线，贯彻“巩固、增强、提升、畅通”八字方针，坚持宏观政策要稳、微观政策要活、社会政策要托底的总体思路，统筹国内国际两个大局，统筹做好稳增长、促改革、调结构、惠民生、防风险、保稳定各项工作，全面做好“六稳”工作，确保各项政策尽快落地见效，努力保持经济平稳健康发展和社会和谐稳定，以优异成绩庆祝中华人民共和国成立70周年。工作中要坚持稳字当头，在发展中化解风险；要保持战略定力，坚定高质量发展方向不动摇；要加快改革创新，激发市场主体活力；要推进高水平开放，拓展合作共赢空间。重点做好以下工作。
　　（一）保持好宏观政策连续性稳定性并适时预调微调。不折不扣实施更大规模减税降费等积极财政政策。切实落实已出台的减税降费政策。加快地方政府专项债券发行和资金使用，更多投向重点领域和重大项目。加大开源节流力度，做好财政收支预算平衡，保障“三保”支出。保持稳健的货币政策松紧适度。深化利率市场化改革，推动小微企业综合融资成本适当下行。督促银行落实好尽职免责和提高不良贷款容忍度要求，引导金融机构增加对制造业、民营企业的中长期融资。深入落实就业优先政策。做好高校毕业生、农民工、退役军人等重点群体就业工作。实施职业技能提升行动，缓解结构性就业矛盾。加快落实高职院校扩招100万人工作。支持以创业带动就业，鼓励农民工返乡创业。
　　（二）着力深化市场化改革扩大高水平开放。持续推进“放管服”改革。大力压减行政许可和整治各类管理措施中的变相审批，推动“双随机、一公开”跨部门联合监管。扎实推进重点领域市场化改革。深化煤电价格、输配电价格改革，全面推广电子不停车快捷收费系统。以共建“一带一路”为引领持续提高开放水平。推动第二届“一带一路”国际合作高峰论坛成果加快落地。积极推动经贸谈判和多双边务实合作。着力稳定外贸增长。落实好稳外贸各项政策措施，完善出口退税政策，扩大出口信用保险覆盖面。积极吸引外商投资。实施2019年版全国和自贸试验区外商投资准入负面清单、[鼓励外商投资产业目录](https://www.pkulaw.com/chl/2e6fab8549042788bdfb.html)，积极推动重大外资项目落地。
　　（三）以改善民生和培育内需新增长点为导向推动形成强大国内市场。合理扩大有效投资。瞄准产业转型升级和关键短板领域持续发力，充分发挥有效投资关键作用。完善地方政府专项债券项目安排协调机制。完善项目资本金管理制度，降低部分基础设施等项目资本金比例。加快制定[政府投资条例](https://www.pkulaw.com/chl/8d88a1cd032de8b9bdfb.html)配套办法。实施城镇老旧小区改造、城市停车场、城乡冷链物流设施等补短板工程，加快推进信息网络等新型基础设施建设。促进消费扩容提质。有序推进老旧汽车报废更新。有效启动农村市场。推进开展普惠养老城企联动专项行动，支持社会力量发展普惠托育服务，开展家政领域培训提升、领跑者、信用建设行动。
　　（四）持续推进创新发展和产业升级。完善创新体制机制和政策，加大知识产权保护力度。推进创新创业创造深入发展。支持打造更多“双创”支撑平台。积极发挥科创板支持科技创新企业发展的作用。着力提升产业基础能力和产业链水平。大力发展战略性新兴产业，大力发展数字经济。坚定不移推进传统产业调整改造提升。加强传统制造业重点领域技术改造。引导金融机构增加对制造业、民营企业的中长期融资。加快“僵尸企业”出清。建立长效机制解决拖欠民营企业、中小企业账款问题。加快发展现代服务业，突破服务业制造业融合发展瓶颈，营造有利于服务业高质量发展的市场环境。
　　（五）统筹推进城乡区域协调发展。扎实推进国家重大区域战略落地。以疏解北京非首都功能为“牛鼻子”推进京津冀协同发展。扎实推进长江经济带生态环境突出问题整改。谋划推动一批长江三角洲区域一体化发展的重点工作和重点项目。加快在海南探索建设中国特色自由贸易港。进一步完善促进区域协调发展的政策体系。完善支持革命老区、民族地区、边疆地区、贫困地区发展的政策。高质量推进新型城镇化建设。推动1亿非户籍人口在城市落户目标取得新进展。加快城市群一体化体制机制建设。扎实推进乡村振兴。以保障粮食安全和重要农产品供给为重点，毫不放松抓好农业生产。加快农村一二三产业融合示范园创建。
　　（六）打好三大攻坚战加快补齐全面建成小康社会短板。加大脱贫攻坚力度，细化落实易地扶贫搬迁后续措施，巩固和拓展脱贫成果。加大污染防治力度。持续推进能源消费总量和强度“双控”，加强饮用水水源地保护工作，有序推进城市生活垃圾分类制度建设。毫不松懈防范重点领域风险。推进金融供给侧结构性改革。完善金融监管法规体系，精准处置好个别中小银行风险，妥善处理公司信用类债券违约事件，把握好风险处置节奏和力度，压实金融机构、地方政府、金融监管部门责任，推动股市平稳健康发展。坚持房子是用来住的、不是用来炒的定位，稳妥实施房地产市场平稳健康发展长效机制方案，不将房地产作为短期刺激经济的手段。
　　（七）以人民为中心做好惠民生各项工作。持续提高民生领域公共服务水平。推进公立医院改革。提前部署今冬明春采暖工作。做好促进居民增收和社会保障相关工作。加大保供稳价力度。压实“米袋子”省长负责制、“菜篮子”市长负责制，加强非洲猪瘟防控、扶持生猪生产。持续提升社会治理水平。深入推进扫黑除恶专项斗争，全力做好防灾减灾救灾工作，持续夯实应急管理工作基础，坚决防范遏制重特大事故。
　　委员长、各位副委员长、秘书长、各位委员，下半年面临的风险挑战有所增多，经济社会发展任务十分繁重，我们要深入学习贯彻习近平新时代中国特色社会主义思想，切实把思想和行动进一步统一到党中央对当前经济形势的判断和工作部署上来。要压实责任、主动作为、敢于担当、真抓实干，坚定不移贯彻党中央、国务院决策部署以及十三届全国人大二次会议要求，自觉接受全国人大的监督，确保各项任务落到实处，促进经济社会持续健康发展。

© 北大法宝：（[www.pkulaw.com](https://www.pkulaw.com/)) 专业提供法律信息、法学知识和法律软件领域各类解决方案。北大法宝为您提供丰富的参考资料，正式引用法规条文时请与标准文本核对。
欢迎查看所有[产品和服务](https://www.pkulaw.com/helps/2.html)。法宝快讯：[如何快速找到您需要的检索结果？](https://www.pkulaw.com/helps/70.html)[法宝](https://www.pkulaw.com/helps/69.html)V6有何新特色？

扫描二维码阅读原文

**原文链接：**<https://www.pkulaw.com/chl/ecfec1f40f2c48bebdfb.html>
